# Increased risk of modality failure with higher serum uric acid level in continuous ambulatory peritoneal dialysis patients: a prospective cohort study

**DOI:** 10.1080/0886022X.2022.2035762

**Published:** 2022-02-16

**Authors:** Shuiqing He, Qianqian Xiong, Li Li, Xuechun Lin, Jing Zhao, Xiaolei Guo, Yuqin He, Wangqun Liang, Chenjiang Ying, Xuezhi Zuo

**Affiliations:** a Department of Clinical Nutrition, Tongji Hospital, Tongji Medical College, Huazhong University of Science and Technology, Wuhan, Hubei, China; b Department of Nutrition and Food Hygiene, Hubei Key Laboratory of Food Nutrition and Safety, School of Public Health, Tongji Medical College, Huazhong University of Science and Technology, Wuhan, Hubei, China; c Department of Nephrology, Tongji Hospital, Tongji Medical College, Huazhong University of Science and Technology, Wuhan, Hubei, China

**Keywords:** CAPD, uric acid, technique failure, mortality, cohort study

## Abstract

**Background:**

Peritoneal dialysis (PD) is one of the most important kidney replacement therapies for patients with end‐stage kidney disease (ESKD). PD technique failure can lead to an escalated cost and increased infectious and cardiovascular risk, up and including to death. The accumulation of uric acid (UA) was associated with adverse outcomes in ESKD patients. However, the relationship between serum UA and technique failure is little explored.

**Methods:**

Here, a total of 266 continuous ambulatory peritoneal dialysis (CAPD) patients (age, 41.8 ± 12.6 years; 125 males) were enrolled and followed up for 31.7 months. Serum UA levels were examined at baseline and each visit. Subjects were divided into three groups according to their baseline serum UA concentrations. Multivariable Cox regression models were used to estimate the hazard ratios (HRs) and 95% confidence intervals (CIs) of PD technique failure.

**Results:**

The level of serum UA increased gradually as time prolonged. During the follow-up period, 77 (28.9%) patients occurred PD technique failure, of which 56 (21.1%) transferred to hemodialysis (HD) and 21 (7.9%) died. Compared to the lowest UA tertile, after adjusting for potential confounders, HRs of technique failure in tertile 2 and tertile 3 were 1.82 (95% CI: 0.95–3.49) and 2.03 (95% CI: 1.05–3.92), respectively, and *p* for trend was 0.043. Adjusted HRs of all-cause technique failure, transferring to HD and mortality with each 1 mg/dL increase in serum UA were 1.20 (95% CI: 1.03–1.40, *p* = 0.019), 1.22 (95% CI: 1.01–1.48, *p* = 0.039), and 1.25 (95% CI: 0.94–1.67, *p* = 0.128), respectively.

**Conclusion:**

Higher serum UA level predicted higher risk of technique failure in CAPD patients.

## Introduction

Peritoneal dialysis (PD) is a cost-effective kidney replacement therapy in end-stage kidney disease (ESKD) patients [1]. Compared to hemodialysis (HD) therapy, PD performed comparable therapeutic effects and may even be superior for patients can schedule treatment time freely, dietary regimens are less restrictive, and longer protection of residual kidney function (RKF) [2,3]. However, the utilization rate of PD is less than 15% of the global dialysis population, and more than 40% peritoneal patients transferred to HD at a median of 21.6 months in a recent study [4]. The high incidence of technique failure may account for the low usage of PD therapy.

Technique failure of PD caused by peritonitis decreased peritoneal solute transfer rate, ultrafiltration failure, circulatory overload, heart failure, or cardiovascular events, finally leading to transferring to HD or even death [5,6]. Undesirably, technique failure of PD incurred greater costs in hospitalization and medication when converted to long-term HD [7], and patients often perceive this transition as a failure, and this notion of failure could carry psychological distress [8]. Moreover, patients transferring to HD most often need a central venous catheter, exposing them to heightened risk of infectious, cardiovascular risk, or even death [9,10]. To date, a number of studies have been conducted to evaluate the risk factors of technique failure. But most of the studies mainly focused on the demographic characteristics, such as age, gender, body mass index, and PD characteristics including peritoneal membrane transport status and ultrafiltration [4,11–13]. Few studies incorporated longitudinal clinical blood biochemical indicators to predict the risk of technique failure in PD patients.

Uric acid (UA), the end product of purine metabolism in humans, is excreted two-thirds by the kidneys [14]. Due to a severe dysfunction of kidneys, most of ESKD patients suffer from hyperuricemia, but commonly manifest asymptomatic hyperuricemia, which has not received enough attention in clinical practice. In PD patients, partial of UA could be eliminated through PD, but the long-term trend of serum UA is unclear. The biological properties of UA as a nephrotoxin with pro-inflammation, oxidative stress, and kidney endothelial dysfunction have been demonstrated in numerous studies [15–18]. Several studies suggested that UA is an independent risk for kidney failure, all-cause mortality, and cardiovascular mortality in PD patients [19], and the hazard ratio per 1 mg/dL higher UA level for all-cause and cardiovascular mortality were 1.33 and 1.44, respectively [20]. However, the relationship between serum UA and technique failure of PD was scarcely reported.

The present longitudinal research was conducted to investigate the long-term trends of serum UA, and explore the relationship between serum UA and PD technique failure in continuous ambulatory peritoneal dialysis (CAPD) patients.

## Materials and methods

### Study population

The PD patients were recruited from a single-center cohort study based on continuous ambulatory peritoneal dialysis patients in central China. During 2013–2020, 266 CAPD patients in Tongji Hospital were recruited with the following select criteria: (1) receiving CAPD treatment longer than 3 months; (2) aged more than 18 years; (3) no diabetic nephropathy; (4) no severe malnutrition (Subjective Global Assessment >15); (5) no diagnosis of gout or receiving urate-lowering drugs; (6) no pregnancy; (7) able to provide consent.

### Ethics

The study was approved by the Institutional Review Board of Tongji Hosipital, Huazhong University of Science and Technology (TJ-IRB20201010), and registered at clinicaltrials.gov (www.chictr.org.cn, registration ID: ChiCTR1900024905). Informed consent was obtained from participants before the study in accordance with the declaration of Helsinki.

### Baseline and follow up

Baseline characteristics of CAPD patients were collected at the first visit after initiation of PD therapy, and patients were followed up every 3 months. Venous blood, 24-h urine, and 24-h spent dialysate were collected at each visit to exam serum UA levels and excretion of UA. All the UA concentrations were measured by auto-biochemistry analyzer machine at the central laboratory of Tongji Hospital at each visit. The daily excretion of UA was urine UA plus with spent dialysate UA. CAPD patients were categorized based on serum UA tertile cutoff points, which were calculated separately in male (Tertile 1 [lowest]: <6.35 mg/dL; Tertile 2 [middle]: 6.35–7.41 mg/dL; Tertile 3 [highest]: >7.41 mg/dL), and female (Tertile 1 [lowest]: <6.01 mg/dL; Tertile 2 [middle]: 6.35–6.98 mg/dL; Tertile 3 [highest]: >6.98 mg/dL). The longitudinal trend of circulatory UA and excretion of UA were investigated over time.

### Covariates assessment

Demographic and comorbidities data, including age, sex, weight, height, systolic blood pressure, diastolic blood pressure, primary kidney disease, edema, hypertension, were collected from the electronic medical records at the initiation of PD. Body mass index (BMI) was calculated as weight divided by height squared (kg/m^2^). Hypertension was recorded if the patient took antihypertensive drugs or had two separate blood pressure measurements ≥140/90 mmHg. Medicine uses of antihypertension drugs, UA lowering drugs, and erythropoietin were recorded according to the prescriptions and adherence of the patient. The biochemical variables including blood urea nitrogen (BUN), serum creatinine, total cholesterol (TC), triglyceride (TG), low-density lipoprotein cholesterol (LDL-C), high-density lipoprotein cholesterol (HDL-C), blood glucose, hypersensitive C-reaction protein (hsCRP), albumin, prealbumin, hemoglobin, potassium, calcium, phosphorus, magnesium, sodium, were measured with auto-biochemistry analyzer machine at the central laboratory of Tongji Hospital.

As described in our previous research, the RKF was calculated as an average of the 24-h urinary urea and creatinine clearances. Weekly total urea clearance (Kt/V) was used to evaluate dialysis adequacy and were calculated from a 24-h collection of dialysate and urine with the use of standard methods, dialysis adequacy was diagnosed as Kt/V ≥ 1.7 [21]. Weekly total creatinine clearance (Ccr) reflected solute clearance rate.

### PD technique failure

**Figure 1.**
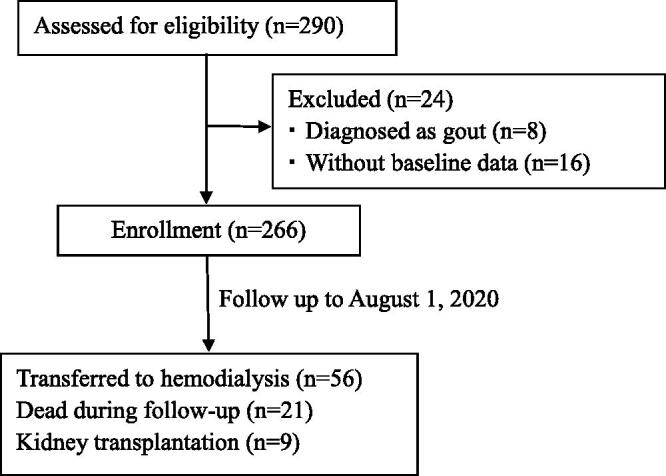
Study flow, including patient enrollment, and outcomes.

Work flow of this cohort study is shown in Figure 1, including patient enrollment and outcomes. The primary outcome of interest was all-cause PD technique failure, including all-cause mortality and transferring to HD. Here in this study, physicians made the decision of the termination of PD therapy when the condition of disease cannot be improved or patients suffered from peritonitis, insufficient clearance of toxin, circulatory overload, heart failure, and other absolute contraindication for PD or even death. Patients would receive HD therapy to maintain favorable survival prognoses, especially when there was no plan for expedited kidney transplantation. Patients with kidney transplantation were censored because they were mainly influenced by economic incomes and suitable donors. All the outcomes were collected exactly through medical records or follow-up information of phone calls. All patients were followed up until transferring to HD therapy, death, kidney transplantation, or censoring on 1 August 2020.

### Statistical analysis

The generalized additive models (GAM) were used to curve fit the trend of UA levels over time in CAPD patients. Participants were stratified into three groups by sex-specific tertiles of baseline serum UA level. Summary statistics are presented as mean ± standard deviation for approximately normally distributed continuous variables, median with interquartile range (IQR) for continuous variables of skewed distribution. Categorical variables were presented as number (*N*) and percentage (%). One-way ANOVA or Kruskal–Wallis test were used to compare differences among three groups for approximately normally distributed continuous variables or skewed distribution variables, respectively. Differences in categorical variables were tested using Chi-square test. A Kaplan–Meier analysis was used to evaluate the change in survival among the tertiles of serum UA, and curves were compared using the log-rank test. Cox proportional hazard regression models were used to investigate the unadjusted and multivariable adjusted associations between serum UA level and PD technique failure. To maximize statistical power to examine the relationship between UA level and PD technique failure, continuous variable analyses were conducted with hazard ratios presented by per 1 mg/dL UA increase. Additionally, Cox regression models were repeated with UA as a continuous variable in stratified analyses to exam for statistical interaction between UA and several confounding factors through multiplicative interaction terms, *p* < 0.1 was considered significant [22]. All statistical analyses were performed using SPSS, version 22.0 (IBM Inc.).

## Results

### Baseline characteristics of patients

A total of 266 CAPD patients (47.0% men and 53.0% women) were enrolled in this prospective cohort study. At baseline, the mean age of the patients was 41.92 years, and the median PD duration was 6.45 (IQR: 4.19–13.95) months. The baseline biochemical parameters were compared among three tertile groups. As shown in Table 1, compared with participants in the lowest serum UA tertile, the higher and highest tertiles showed worse residual kidney function. Moreover, patients in the higher serum UA tertile had higher level of TG, phosphorus, sodium, and lower level of HDL-C. According to the complications and drugs records, most of the patients encompassed hypertension (84.2%), and 69.5% of patients took antihypertension drugs.

**Table 1.** Baseline characteristics according to tertiles of serum UA level.

	Overall (*N* = 266)	Tertile 1 (*n* = 85)	Tertile 2 (*n* = 93)	Tertile 3 (*n* = 88)	*p*
Demographics					
Gender (M/F)	125/141	39/46	45/48	41/47	0.942
Age (year)	41.77 ± 12.56	40.01 ± 12.96	42.26 ± 12.25	43.01 ± 12.43	0.371
Dialysis duration (mo)	6.45 (4.19–13.95)	6.70 (4.30–12.97)	6.23 (3.97–14.73)	7.42 (4.24–14.98)	0.821
BMI (kg/m^2^)	21.12 ± 2.89	20.65 ± 2.33	21.08 ± 2.85	21.62 ± 3.34	0.089
SBP (mmHg)	148.35 ± 22.20	148.68 ± 21.02	148.73 ± 22.35	147.64 ± 23.38	0.935
DBP (mmHg)	91.23 ± 14.99	92.97 ± 13.47	91.04 ± 15.77	89.69 ± 15.57	0.397
Renal function					
Serum creatinine (μmol/L)	917.00 (780.75–1106.50)	875.00 (750.00-1067.00)	945.00 (836.00-1133.00)	909.00 (756.75-1145.75)	0.120
BUN (mmol/L)	17.99 (14.19–24.05)	17.51 (12.84–24.37)	16.67 (13.70–23.65)	19.08 (16.47–24.15)	0.063
RKF (mL/min·1.73 m^-2^)	4.64 (3.87–5.81)	4.75(4.13–6.32)	4.32 (3.57–5.43)	4.70 (3.47–5.75)	0.010
Urine volume (mL)	700.00 (300.00–1000.00)	500.00 (200.00–1012.50)	700.00 (300.00–950.00)	750.00 (500.00–1100.00)	0.048
Dialysis adequacy					
Ccr (L/week·1.73 m^-2^)	64.34 ± 18.30	65.22 ± 15.28	64.02 ± 20.51	63.79 ± 18.79	0.754
Kt/V	2.02 ± 0.51	2.08 ± 0.42	2.05 ± 0.66	1.92 ± 0.39	0.072
Dialysate effusion (L)	7.21 ± 1.63	7.61 ± 1.46	7.23 ± 1.48	6.79 ± 1.84	0.008
page range					
TC (mmol/L)	4.69 ± 1.08	4.68 ± 1.19	4.74 ± 1.08	4.65 ± 0.97	0.880
TG (mmol/L)	1.61 ± 0.98	1.45 ± 0.62	1.58 ± 1.02	1.81 ± 1.18	0.049
LDL-C (mmol/L)	2.62 ± 0.77	2.63 ± 0.82	2.65 ± 0.77	2.59 ± 0.72	0.814
HDL-C (mmol/L)	1.18 ± 0.33	1.23 ± 0.33	1.21 ± 0.33	1.11 ± 0.31	0.027
Glucose (mmol/L)	5.53 ± 1.24	5.64 ± 1.64	5.46 ± 0.99	5.50 ± 1.00	0.844
hs-CRP (mg/L)	1.10 (0.40–2.98)	0.90 (0.30–3.50)	1.00 (0.48–1.95)	1.40 (0.50–4.20)	0.170
Serum UA (mg/dL)	6.86 ± 1.51	5.42 ± 0.77	6.71 ± 0.35	8.44 ± 1.27	<0.001
Urine UA (μmol/L)	345.00 (183.00–519.50)	269.00 (163.00–427.00)	332.00 (156.50–423.00)	526.50 (288.00–714.90)	<0.001
Dialysate UA (μmol/L)	139.90 ± 51.00	122.18 ± 37.60	143.99 ± 50.68	154.90 ± 58.26	<0.001
Daily UA excretion (mg/d)	224.33 ± 102.14	195.64 ± 65.90	216.59 ± 68.02	262.06 ± 142.44	0.001
Nutritional status					
Albumin (g/L)	38.64 ± 4.56	38.40 ± 5.10	38.51 ± 4.32	39.00 ± 4.24	0.651
Prealbumin (mg/L)	390.55 ± 74.17	386.43 ± 74.08	399.79 ± 79.58	386.08 ± 68.89	0.572
Hemoglobin (g/L)	101.67 ± 22.91	100.03 ± 22.43	103.21 ± 22.86	101.70 ± 23.56	0.809
Potassium (mmol/L)	4.38 ± 0.73	4.26 ± 0.76	4.50 ± 0.79	4.37 ± 0.61	0.071
Corrected calcium (mmol/L)	2.39 ± 0.22	2.40 ± 0.19	2.39 ± 0.23	2.37 ± 0.25	0.599
Phosphorus (mmol/L)	1.65 ± 0.53	1.51 ± 0.56	1.70 ± 0.51	1.75 ± 0.50	0.006
Magnesium (mmol/L)	0.89 ± 0.15	0.87 ± 0.13	0.89 ± 0.16	0.91 ± 0.16	0.085
Comorbidities and medications					
Edema (%)	166 (62.4)	55 (64.7)	59 (63.4)	52 (59.1)	0.724
Hypertension (%)	224 (84.2)	75 (88.2)	76 (81.7)	73 (83.0)	0.455
Antihypertensive drugs (%)	185 (69.5)	57 (67.1)	63 (67.7)	65 (73.9)	0.558
Erythropoietin (%)	168 (74.3)	57 (74.0)	56 (73.7)	55 (75.3)	0.971

Continuous variables are expressed as mean ± standard deviation or median (interquartile range), categorical variables are given as number (percentage).

*p* Value comparisons across continuous variables are based on one-way ANOVA or Kruskal–Wallis test; *p* value comparisons across categorical variables are based on chi-square test or Fisher’s exact test. BMI: body mass index; SBP: systolic blood pressure; DBP: diastolic blood pressure; BUN: blood urea nitrogen; RKF: residual kidney function; Ccr: creatinine clearance rate; Kt/V: weekly total urea clearance; TC: total cholesterol; TG: triglyceride; LDL-C: low-density lipoprotein cholesterol; HDL-C: high-density lipoprotein cholesterol; hs-CRP: hypersensitive C-reaction protein.

### Long-term trend of uric acid in CAPD patients

**Figure 2.**
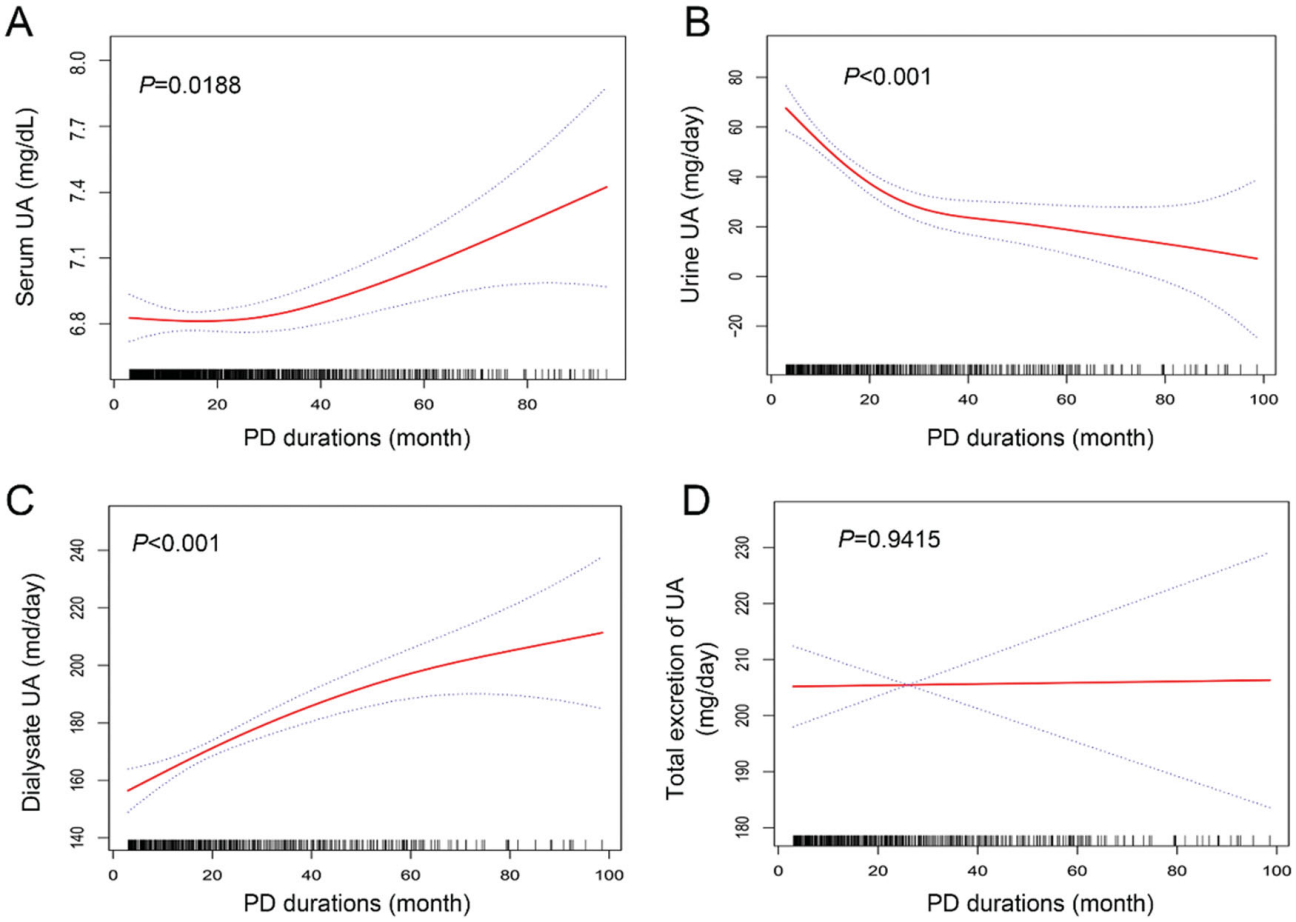
The long-term trends of serum UA and excretion of UA in CAPD patients. *p* Values were analyzed by using generalized additive models to evaluate the fluctuations over time.

The longitudinal trend of circulatory UA and excretion of UA are shown in Figure 2. The results of curve fitting with generalized additive model indicated the level of serum UA increased gradually as the dialysis time prolonged. The daily excretion of UA through urine decreased gradually, while the dialysate UA increased over time. Taken together, the total excretion of UA tends to be a stable state with the extension of dialysis time. Nevertheless, only 200–220 mg UA was eliminated through RKF and peritoneal dialysis therapy each day.

### Association between baseline serum UA and PD technique failure

**Figure 3.**
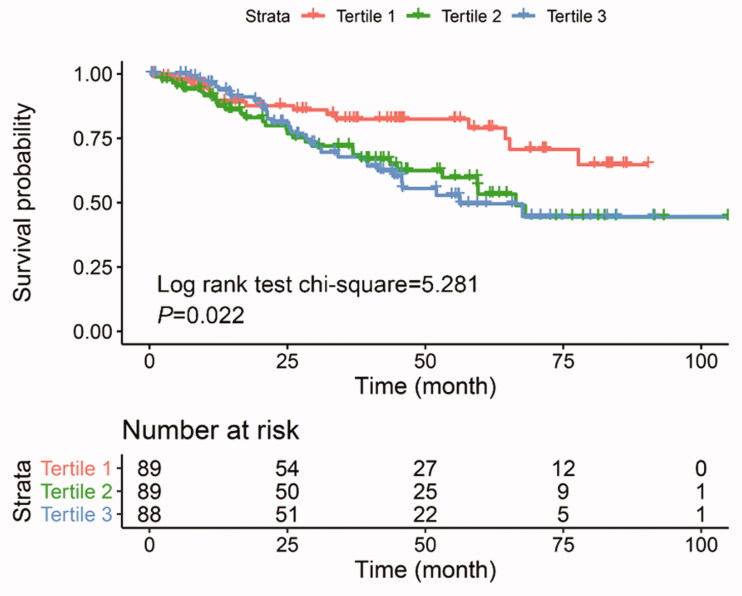
Kaplan–Meier survival curves estimate the risk of PD failure between sex-specific tertiles of serum UA levels.

The median follow-up period was 31.7 months (IQR: 12.1–55.4 months). During the follow-up period, 56 CAPD patients were transferred to HD therapy, 21 patients occurred death, of which 10 patients (47.6%) died of cardiovascular events. Overall, the incidence rate of PD technique failure was 28.9%. According to the results of the univariate and multivariate Cox regression models (shown in Supplementary Table S1), lower serum albumin, higher serum UA, and lower RKF were associated with technique failure in CAPD patients, while the hazard ratios (HRs) and 95% confidence intervals were 0.91 (0.86–0.96), 1.18 (1.03–1.36), and 0.48 (0.25–0.93), respectively. The Kaplan–Meier survival curves for PD technique failure in patients with different serum UA levels are illustrated in Figure 3. Compared with participants in the lowest serum UA tertile, the risk for PD technique failure in the higher UA level groups significantly increased (log-rank test *p* = 0.022).

Table 2 displays the HRs of PD technique failure that was associated with different UA levels while considering tertile 1 group as the reference. After adjusting for various covariates, the HR and 95% confidence intervals for PD technical failure in tertile 2 and tertile 3 were 1.82 (0.95–3.49) and 2.03 (1.05–3.92), respectively, and *p* value for trend was 0.043. Compared with tertile 1 group, the highest sex-specific tertile (tertile 3) of serum UA concentration was associated with a higher risk of transferring to HD therapy, and *p* value for trend was 0.043. The risk of mortality was higher in tertile 2 compared to tertile 1 group in multivariable Cox regression model 3, but without significance.

**Table 2.** Hazard ratios of PD technique failure for sex-specific tertiles of UA level.

	Tertile 1 (*n* = 85)	Tertile 2 (*n* = 93)	Tertile 3 (*n* = 88)	*p* For trend
All-cause PD technique failure				
*N* (%)	16 (18.0%)	30 (33.7%)	31 (35.2%)	0.011
Unadjusted	1.0 (Reference)	2.01 (1.10–3.70)	2.07 (1.13–3.78)	0.023
Model 1	1.0 (Reference)	2.14 (1.15–3.96)	2.13 (1.15–3.93)	0.021
Model 2	1.0 (Reference)	1.75 (0.92–3.31)	1.86 (0.99–3.48)	0.064
Model 3	1.0 (Reference)	1.86 (0.97–3.54)	2.06 (1.08–3.92)	0.034
Transferred to hemodialysis				
*N* (%)	12 (14.1%)	20 (25.3%)	24 (29.6%)	0.017
Unadjusted	1.0 (Reference)	1.85 (0.91–3.80)	2.19 (1.09–4.40)	0.028
Model 1	1.0 (Reference)	1.95 (0.94–4.05)	2.24 (1.11–4.52)	0.028
Model 2	1.0 (Reference)	1.66 (0.73–3.46)	2.05 (1.00–4.20)	0.050
Model 3	1.0 (Reference)	1.74 (0.81–3.76)	2.30 (1.09–4.84)	0.029
Death				
*N* (%)	4 (5.2%)	10 (14.5%)	7 (10.9%)	0.227
Unadjusted	1.0 (Reference)	2.93 (0.92–9.37)	2.12 (0.62–7.25)	0.252
Model 1	1.0 (Reference)	3.51 (1.08–11.41)	2.21 (0.62–7.88)	0.233
Model 2	1.0 (Reference)	2.67 (0.76–9.37)	1.74 (0.46–6.56)	0.498
Model 3	1.0 (Reference)	3.09 (0.88–10.81)	1.98 (0.53–7.37)	0.378

Hazard ratios (95% confidence interval) and *p* values were analyzed by Cox regression models. Model 1 is adjusted for age, sex, BMI, dialysis durations. Model 2 is adjusted for Model 1 covariates and log-transformed RKF, log-transformed BUN, Kt/V. Model 3 is adjusted for Model 2 covariates and albumin, phosphorus, edema, hypertension, antihypertensive drugs.

Furthermore, serum UA was examined as a continuous variable. As shown in Table 3, according to the Cox proportional hazards model, higher serum UA level were associated with higher risk of PD technique failure with or without adjustment for confounding factors. Adjusted HRs of PD technique failure, transferring to HD and mortality with each 1 mg/dL increase in serum UA were 1.20 (95% CI: 1.03–1.40, *p* = 0.019), 1.22 (95% CI: 1.01–1.48, *p* = 0.039), and 1.25 (95% CI: 0.94–1.67, *p* = 0.128), respectively.

**Table 3.** Hazard ratios of PD technique failure for each 1 mg/dL serum UA increase.

	All-cause PD technique failure		Transferred to hemodialysis		Death	
	HR (95% CI)	*p*	HR (95% CI)	*p*	HR (95% CI)	*p*
Unadjusted	1.21 (1.06–1.39)	0.005	1.22 (1.04–1.44)	0.018	1.29 (1.02–1.65)	0.037
Model 1	1.22 (1.06–1.40)	0.005	1.22 (1.03–1.45)	0.020	1.32 (1.02–1.70)	0.033
Model 2	1.19 (1.03–1.38)	0.021	1.20 (1.01–1.43)	0.041	1.25 (0.94–1.65)	0.123
Model 3	1.20 (1.03–1.39)	0.018	1.23 (1.02–1.48)	0.032	1.21 (0.91–1.59)	0.185

Hazard ratios (95% confidence interval) and *p* values were analyzed by Cox regression models. Model 1 is adjusted for age, sex, BMI, dialysis durations. Model 2 is adjusted for Model 1 covariates and log-transformed RKF, log-transformed BUN, Kt/V. Model 3 is adjusted for Model 2 covariates and albumin, phosphorus, edema, hypertension, antihypertensive drugs.

The effect of high serum UA on PD technique failure was further performed by stratified analysis. The entire cohort was divided to subgroups by gender or the median value of age, BMI, PD duration, albumin, RKF, and Kt/V. As shown in Table 4, the risk of PD technique failure per 1 mg/dL increase in UA concentration was most prominent in lower RKF group (*p* for interaction was 0.013). All other factors included in the analysis showed nonsignificant interactions.

**Table 4.** Stratified analyses by various confounder factors.

	Events/*N*	HR (95% CI)	*p* For interaction
Age (years)			
≤43	31/138	1.22 (0.93–1.60)	0.137
>43	46/128	1.20 (1.00–1.44)	
Gender			
Male	41/125	1.14 (0.88–1.48)	0.185
Female	36/141	1.27 (1.04–1.55)	
BMI (kg/m^2)^			
≤21.0	35/136	1.40 (1.13–1.75)	0.188
>21.0	42/130	1.07 (0.85–1.34)	
PD durations (months)			
≤6.4	40/136	1.06 (0.83–1.34)	0.653
>6.4	37/130	1.27 (1.03–1.57)	
Albumin (g/L)			
≤39.0	44/134	1.05 (0.85–1.30)	0.229
>39.0	33/132	1.37 (1.04–1.80)	
RRF (mL/min·1.73 m^-2^)			
≤4.64	38/133	1.35 (1.09–1.67)	0.013
>4.64	39/133	1.13 (0.89–1.43)	
Kt/V			
≤1.98	48/133	1.12 (0.92–1.36)	0.736
>1.98	29/133	1.32 (1.03–1.72)	

The median values of these parameters were used to divide the entire cohort into two subgroups. Hazard ratios (95% confidence interval) were analyzed by Cox regression models and adjusted for confounders as Model 3 in Table 3. The multiplicative interaction terms were included in models to detect the interaction effect. BMI: body mass index; RKF: residual kidney function; Kt/V.

## Discussion

In the present study, we observed the level of serum UA increased gradually as the peritoneal dialysis time prolonged while the total excretion of UA tended to be stable. Furthermore, higher serum UA level at baseline predicted a higher risk for PD technique failure.

Since a large part of UA is excreted through kidneys, it is conceivable that decreased kidney function is accompanied by increased serum UA. PD therapy is used to eliminate wastes and toxins accumulated in ESKD patients. However, our results indicated that the serum UA of CAPD patients elevated gradually over time. The decreased urine UA possibly attributed to the declined RKF as the dialysis time prolonged. In total, only 200–250 mg/day UA was eliminated in CAPD patients, even though the dialysate excretion of UA increased over time. The excretion of UA in CAPD patients was far less than 500–750 mg/day through urine in healthy person or more in hyperuricemia patients with normal kidney function [23,24]. The results suggested that the insufficient elimination of UA and increasing serum UA in CAPD patients cannot be neglected.

Patients with higher UA level were associated with higher phosphorus, triglyceride and lower HDL-C in the present study. It was similar to Xiang’s report that they found higher serum UA level was independently associated with higher serum albumin, higher BMI, higher phosphorus concentrations, which reflected nutritional status [19]. Additionally, hyperuricemia is also associated with hypertriglyceridemia and metabolic syndrome in chronic kidney disease (CKD), which implies undesirable outcomes may occur in CKD patients [25].A handful of studies have described the relationship between serum UA and adverse outcomes among PD individuals [26,27]. Xiang et al. [19] conducted a large multicenter observational study and found that a higher UA level (≥7.28 mg/dL) was significantly associated with higher risk of all-cause mortality, independent of several confounding factors. A very recent paper by Yoshida et al. [28] demonstrated increased risk of infection (at least in HD patients) with elevated UA. Another epidemiologic study in a large-scale registry of Japanese PD patients revealed a U-shaped relationship between serum UA level and all-cause mortality, that both lower (<5.0 mg/dL) and higher (8.0 mg/dL) UA level were independently associated with higher all-cause mortality [29]. Here, this study focused not only on mortality but also on the composite outcome in PD patients (including transferring to HD and mortality). Our data indicated that elevated serum UA was independently associated with all-cause technique failure in CAPD patients, which was consistent with Hsieh’s report that serum UA was independently associated with peritonitis-related technique failure [30]. The conclusions were controversial that an inverse relationship between hyperuricemia and mortality in CAPD patients was demonstrated in a retrospective study [31], and decreased serum UA in the follow-up predicted higher all-cause mortality of PD patients [32]. Here, the relationship between UA and all-cause mortality was no longer significant after the adjustment of potential confounders. The exact reasons for these inconsistent relationship between serum UA level and poor outcomes remain to be clarified. On the one hand, elevated UA act as a kidney toxin with the capability of pro-inflammation, oxidative stress, and kidney endothelial dysfunction, which could lead to adverse outcomes [16]. On the other hand, lower serum UA level may reflect the malnourishment status because major source of UA is derived from dietary intake of purines and nucleotides [31]. The malnourishment status in turn accounts for susceptibility to adverse outcomes. Here in this study, severe malnutritional patients were excluded in this cohort study, and Cox regression models were adjusted for BMI and serum albumin which were confounders of nutritional status.

Additionally, here in this cohort study, patients with diabetic nephropathy were excluded, the results suggested that baseline elevated serum UA level were associated with a higher risk of technique failure in non-diabetic PD patients, which was similar to Xia’s findings that higher serum UA level predicted higher risk of all-cause mortality in non-diabetic PD patients, but no significant association was found in female diabetic PD patients [33]. It implied that there may be complicated confounders in diabetes nephropathy patients, and the relationship between UA level and technique failure in diabetic PD patients needed more investigations. Besides, the rate of reduction in RKF was also significantly associated with hyperuricemia [34]. Similarly, the risk of progressive RRF loss with hyperuricemia was also reported by Yang et al. [35]. RKF, Kt/V, and Ccr were regarded as important confounders and adjusted in regression models, the results indicated serum UA independently associated with risk of technique failure. And significant interaction effect between serum UA and RKF was observed, the risk of PD technique failure for higher UA level was more prominent in lower RKF group. The RKF and peritoneal dialysis adequacy were widely reported as a strong predictor of technique survival and mortality among PD patients [36,37]. Thus, it was no surprise that the lower RKF aggravated the adverse effect of high level of UA on technique failure.

The underlying mechanisms connecting elevated serum UA level with higher risk of PD technique failure are still far from being well understood. Technique failure can occur for a number of reasons, including peritonitis, inadequate dialysis, ultrafiltration failure, circulatory overload, heart failure, or cardiovascular events. Peritonitis is the leading cause of technique failure and accounts for nearly half of the failures [38,39]. Uric acid crystals or soluble urate activate the NLRP3 inflammasome and present pro-inflammatory properties and can thus contribute to the state of chronic low-grade inflammation, which is a widely accepted pathogenic mechanism in several pathologies [40,41]. Additionally, the elevated serum UA could cause imbalances in oxidation and antioxidant, and promote the formation of reactive oxygen species, which can impact the immune response and lead to inflammation, or even contribute to peritoneal fibrosis [42,43]. Meanwhile, PD technique failure caused by inadequate dialysis and ultrafiltration failure are mainly imputed to the structural and functional change of peritoneum [44,45]. Besides, high UA level induce proximal tubular dysfunction and endothelial dysfunction, reduce endothelial nitric oxide bioavailability, and lead to maladaptive changes in glomeruli and the tubulointerstitium, which would damage the residual kidney function [46]. Actually, there are too many causes for PD technique failure, and the power of test needs to be improved in cohort study with larger sample size. The different effects of hyperuricemia on each specific cause needed to be investigated yet.

The strengths of our study are as follows: (1) the prospective cohort study provided longitudinal changes in UA level and a gradually rising trend in serum UA was observed in continuous PD patients; (2) our results remained robust after several stratification analysis and adjustments for multiple potential confounders including demographic characteristics, laboratory data, nutritional status, comorbidities, and medications; (3) patients with complicated comorbidities like diabetes and diabetic nephropathy were excluded to avoid the potentially confounding factors associated with poor outcomes. There are also several limitations in this study: (1) it was conducted in a single center and had a relatively small sample size, the number of death events were relatively limited, and the statistical power was insufficient to draw a final conclusion about the association between serum UA and mortality in these patients; (2) only CAPD patients without diabetes were recruited in this study, caution should be taken when generalizing the conclusion to other populations; (3) as an observational study, the associations do not prove causality, the underlying mechanisms linking elevated serum UA and technique failure needed further clarification.

In conclusion, higher serum UA concentration was associated with higher risk of technique failure in CAPD patients. It is advisable to integrate serum UA and other clinical characteristics to adjust therapeutic program in advance to prevent worse outcomes. Further studies are needed to explore whether UA-lowering treatment improve technique survival in CAPD patients, and to clarify the underlying mechanisms.

## Supplementary Material

Supplemental Material

## Data Availability

The data that support the findings of this study are not publicly available due to their containing information that could compromise the privacy of research participants but are available from the corresponding authors [Zuo X], zuo1967@tjh.tjmu.edu.cn and [Ying C], yingcj@hust.edu.cn.
