# Supplementary material for: Increased risk of modality failure with higher serum uric acid level in continuous ambulatory peritoneal dialysis patients: a prospective cohort study
Source: Ren Fail. 2022 Feb 16;44(1):272–81. doi: 10.1080/0886022X.2022.2035762 (PMC8856069; doi:10.1080/0886022X.2022.2035762)
Supplement: Supplemental Material [file IRNF_A_2035762_SM3692.pdf]

Table S1 Risk factors for technique failure in PD patients.

	Univariate		Multivariate	
	HR (95% CI)	*P* value	HR (95% CI)	*P* value
Age (per 5 years)	1.12 (1.02-1.22)	0.015	-	-
Sex (Male=1, Female=2)	0.82 (0.51-1.26)	0.335	-	-
Dialysis durations (per 6 months)	0.98 (0.91-1.07)	0.704	-	-
BMI (per 1 kg/m^{-2})	1.04 (0.97-1.13)	0.292	-	-
SBP (per 10 mmHg)	1.05 (0.95-1.16)	0.357	-	-
DBP (per 10 mmHg)	0.98 (0.85-1.14)	0.807	-	-
Albumin (per 1 g/L)	0.92 (0.87-0.96)	<0.001	0.91 (0.86-0.96)	<0.001
Hemoglobin (per 1 g/dL)	0.95 (0.86-1.05)	0.336	-	-
Potassium (per 1 mmol/L)	0.94 (0.69-1.28)	0.676	-	-
Corrected calcium (per 1 mmol/L)	0.65 (0.25-1.71)	0.385	-	-
Phosphorus (per 1 mmol/L)	1.44 (0.98-2.11)	0.066	-	-
Magnesium (per 1 mmol/L)	0.55 (1.12-2.50)	0.437	-	-
Creatinine (per 1 mg/dL)	1.06 (0.99-1.13)	0.084	-	-
lnBUN (per 1 mmol/L)	1.24 (0.87-1.77)	0.233	-	-
Serum UA (per 1 mg/dL)	1.21 (1.06-1.39)	0.005	1.18 (1.03-1.36)	0.019
TC (per 1 mmol/L)	1.19 (0.95-1.48)	0.128	-	-
TG (per 1 mmol/L)	1.07 (0.87-1.33)	0.514	-	-
LDL-C (per 1 mmol/L)	1.31 (0.97-1.77)	0.080	-	-
HDL-C (per 1 mmol/L)	0.75 (0.35-1.63)	0.471	-	-
Blood glucose (per 1 mmol/L)	0.97 (0.82-1.16)	0.766	-	-
lnRKF (per 1 mL·min^{-1}·1.73 m^{-2})	0.41 (0.21-0.81)	0.010	0.48 (0.25-0.93)	0.030
Kt/V (per 1 L·wk^{-1}·1.73 m^{-2})	0.47 (0.28-0.81)	0.007	-	-
Ccr (per 10 L·wk^{-1}·1.73 m^{-2})	0.89 (0.78-1.01)	0.078	-	-
Edema (Yes=1, No=0)	1.35 (0.85-2.15)	0.198	-	-
Hypertension (Yes=1, No=0)	0.77 (0.43-1.35)	0.354	-	-
Antihypertensive (Yes=1, No=0)	0.85 (0.53-1.37)	0.507	-	-

Abbreviations: BMI, body mass index; SBP, systolic blood pressure; DBP, diastolic blood pressure; lnBUN, log-transformed residual renal function blood urea nitrogen; TC, total cholesterol; TG, triglyceride; LDL-C, low-density lipoprotein cholesterol; HDL-C, high-density lipoprotein cholesterol; lnRKF, log-transformed residual kidney function; Ccr, creatinine clearance rate; Kt/V, weekly total urea clearance;

Table S2. Hazard ratios of PD technique failure for each 1 mg/dL serum UA increase in 276 PD patients [a].

	All-cause PD technique failure		Transferred to hemodialysis		Death	
	HR (95% CI)	*P*	HR (95% CI)	*P*	HR (95% CI)	*P*
unadjusted	1.23 (1.08-1.40)	0.002	1.25 (1.06-1.46)	0.007	1.30 (1.02-1.66)	0.033
Model 1	1.24 (1.08-1.42)	0.002	1.25 (1.06-1.47)	0.008	1.33 (1.03-1.71)	0.028
Model 2	1.22 (1.06-1.41)	0.007	1.27 (1.07-1.51)	0.007	1.23 (0.93-1.62)	0.141
Model 3	1.23 (1.06-1.43)	0.005	1.28 (1.06-1.53)	0.009	1.26 (0.95-1.68)	0.115

[a] patients with gout or receiving urate-lowering drugs were included. Hazard ratios (95% confidence interval) and *P* values were analyzed by cox regression models.

Model 1 is adjusted for age, sex, body mass index (BMI), dialysis durations.

Model 2 is adjusted for model 1 covariates and log-transformed RKF, log-transformed BUN, Kt/V albumin, phosphorus, TG, HDL-C.

Model 3 is adjusted for model 2 covariates and edema, hypertension, antihypertensive drugs.
